# Computer programs used in the field of hospital pharmacy for the management of dangerous drugs: systematic review of literature

**DOI:** 10.3389/fpubh.2023.1233264

**Published:** 2023-08-30

**Authors:** Seira Climent-Ballester, Pedro García-Salom, Javier Sanz-Valero

**Affiliations:** ^1^Pharmacy Service, Dr. Balmis General University Hospital, Alicante Institute for Health and Biomedical Research (ISABIAL), Alicante, Spain; ^2^Pharmacy Service, Dr. Balmis General University Hospital, Alicante, Spain; ^3^National School of Occupational Medicine, Carlos III Health Institute, Madrid, Spain

**Keywords:** antineoplastic agents, cytostatic agents, hazardous substances, medical informatics applications, software, Hospital Pharmacy, patient safety, occupational health

## Abstract

**Background:**

This review wants to highlight the importance of computer programs used to control the steps in the management of dangerous drugs. It must be taken into account that there are phases in the process of handling dangerous medicines in pharmacy services that pose a risk to the healthcare personnel who handle them. Objective: To review the scientific literature to determine what computer programs have been used in the field of hospital pharmacy for the management of dangerous drugs (HDs).

**Methods:**

The following electronic databases were searched from inception to July 30, 2021: MEDLINE (via PubMed), Embase, Cochrane Library, Scopus, Web of Science, Latin American and Caribbean Literature in Health Sciences (LILACS) and Medicine in Spanish (MEDES). The following terms were used in the search strategy: “Antineoplastic Agents,” “Cytostatic Agents,” “Hazardous Substances,” “Medical Informatics Applications,” “Mobile Applications,” “Software,” “Software Design,” and “Pharmacy Service, Hospital.”

**Results:**

A total of 104 studies were retrieved form the databases, and 18 additional studies were obtained by manually searching the reference lists of the included studies and by consulting experts. Once the inclusion and exclusion criteria were applied, 26 studies were ultimately included in this review. Most of the applications described in the included studies were used for the management of antineoplastic drugs. The most commonly controlled stage was electronic prescription; 18 studies and 7 interventions carried out in the preparation stage focused on evaluating the accuracy of chemotherapy preparations.

**Conclusion:**

Antineoplastic electronic prescription software was the most widely implemented software at the hospital level. No software was found to control the entire HD process. Only one of the selected studies measured safety events in workers who handle HDs. Moreover, health personnel were found to be satisfied with the implementation of this type of technology for daily work with these medications. All studies reviewed herein considered patient safety as their final objective. However, none of the studies evaluated the risk of HD exposure among workers.

## 1. Introduction

In 1979, Falck et al. (1) detected an elevated level of cellular mutagenicity in the urine of nurses who worked with cytostatic drugs; subsequently, there has been a growing concern regarding the safe handling of hazardous substances among health workers.

The concept of “hazardous drugs” (HDs), which was previously associated exclusively with cytostatic drugs, was introduced in 1990 by the American Society of Hospital Pharmacists (ASHP) and adopted in 2004 by the National Institute for Occupational Safety and Health (NIOSH), thus giving rise to the current internationally accepted definition: “any drug that presents in humans one or more of the following hazard criteria: carcinogenicity, teratogenicity or other developmental toxicity, reproductive toxicity, organ toxicity at low doses, genotoxicity or those drugs with a structure or toxicity profile similar to other dangerous drugs” (2).

In 2014, the NIOSH classified HDs into three groups: antineoplastic drugs; nonantineoplastic drugs, with some criteria of danger; and drugs that present risk for the reproductive process, pregnant or lactating women but do not carry risk for the rest of the staff (3).

Since this classification, numerous studies have shown that HDs carry chemical risks for workers who handle them (4). However, recent efforts have focused on controlling the management of HDs to guarantee patient safety, i.e., the prescription, validation and administration of drugs, as well as avoiding potentially harmful medication-related errors. Technological tools have been very helpful for improving the safety of HDs and enhancing the efficiency of the system for managing these drugs, including the advent of electronic prescriptions, the identification of drugs with different types of codes and the use of intelligent infusion pumps (5).

The complexity and interdisciplinary nature of the HD manipulation process make it especially vulnerable to errors. Therefore, HDs are considered a high-risk therapy and can have serious sequelae for both the patient and the professional who handles them (6).

Efforts have been made to establish guidelines that guarantee the safe use of HD; however, there is no global consensus with respect to standards for preventing HD exposure (7).

Therefore, it is essential to standardize the processes, since when a protocol is based on clinical guidelines, variability decreases, thus improving quality and minimizing the risks associated with HD management (8).

Risk assessment plays a key role in the management and control of the PM process, since all measures that are adopted to guarantee the safety of the process are based on its results. A recent study on the perceived risk of exposure in the management of HDs in hospital units (9) recommended integrating HDs into a standardized management system to improve the safety of drugs; this type of risk management model is applicable to any health center.

Although such risk analyses are usually required (4) and there is a well-known need for them, few studies have described risk analyses for HDs; to date, the Hazardous Drug Consensus Group has established the only concrete methodological proposal to carry out a risk analysis with respect to HDs (10).

Therefore, it is essential to determine the risks associated with the HD management process, obtain the necessary information to adopt preventive measures, and minimize risks. It is essential to identify the main stages and operations of the HD management process as well as the preventive measures that can be applied to avoid occupational exposure to HDs (2). Previous studies have examined the hazards and risks associated with this process (4, 11) and the perception of the magnitude of the risk. The evaluation of the different parameters related to risk is especially important for the standardization of behaviors and practices in the area, with the aim of establishing a rational policy to protect the health of workers (12).

Due to the computerization of the processes, it is possible to obtain a considerable amount of information on the stages and operations of the HD manipulation process and obtain a (historical) report of current practices. Additionally, this type of analysis will enable us to identify risks and develop preventive measures that guarantee the safety of the whole process. Thus, the implementation and development of specific computer programs for the comprehensive management of HDs aims to reduce the risks associated with the manipulation of these substances.

Therefore, taking into account that technology has been shown to be very helpful in the management of HDs, the objective of this study was to review the scientific literature to determine which computer programs have been used in the field of hospital pharmacy for managing potential exposure to HDs. And in this way, obtain a point of reference on the current situation of workers who handle HD in hospital pharmacy services, as well as the preventive measures implemented through the computerization of processes and their evaluation. To achieve this objective, a systematic technique was used.

## 2. Materials and methods

### 2.1. Design

The current study used a cross-sectional descriptive design and a critical analysis of previous studies retrieved through systematic review. The structure of this review followed the Preferred Reporting Items for Systematic Reviews and Meta-Analyses (PRISMA) guidelines and the methodological framework proposed by Arksey and O’Malley (13) for scoping studies. Likewise, the methodological design of Arenas-Escaso et al. (14) was taken into consideration.

### 2.2. Source of data collection

The following electronic databases were searched: MEDLINE (via PubMed), Embase, Cochrane Library, Scopus, Web of Science, Latin American & Caribbean Health Sciences Literature (LILACS) and Medicine in Spanish (MEDES).

### 2.3. Unit of analysis

We examined the articles retrieved from the abovementioned databases.

### 2.4. Information processing

To select the search terms, we used the Thesaurus of Descriptors in Health Sciences (DeCS), which was developed by the Latin American and Caribbean Center for Medical Sciences Information (BIREME), and Medical Subject Headings (MeSH) terms, which were developed by the US National Library of Medicine.

Based on the hierarchy of both the thesaurus and the indexing files, the following search equations were considered adequate:

- Equation 1 - Dangerous drug.

“Antineoplastic Agents”(Mesh) OR “Antineoplastic Agent*”(Title/Abstract) OR “Antineoplastic Drug*”(Title/Abstract) OR “Antineoplastic*”(Title/Abstract) OR “Chemotherapeutic Anticancer Drug*”(Title/Abstract) OR “Antitumor Drug*”(Title/Abstract) OR “Cancer Chemotherapy Agent*”(Title/Abstract) OR “Cancer Chemotherapy Drug*”(Title/Abstract) OR “Chemotherapeutic Anticancer Agent*”(Title/Abstract) OR “Anticancer Agent*”(Title/Abstract) OR “Antitumor Agent*”(Title/Abstract) OR “Hazardous Substances”(Mesh) OR “Hazardous Material*”(Title/Abstract) OR “Hazardous Chemical*”(Title/Abstract) OR “Environmental Toxic Substance*”(Title/Abstract) OR “Toxic Environmental Substance*”(Title/Abstract) OR “Biohazard*”(Title/Abstract) OR “Cytostatic Agents”(MeSH Terms) OR “Cytostatic Agents*”(Title/Abstract) OR “Cytostatic*”(Title/Abstract) OR “Cytostatic Drug*”(Title/Abstract) OR “Hazardous Drug*”(Title/Abstract) OR “Chemotherapy”(Title/Abstract) OR “Chemotherapeutic Agent*”(Title/Abstract) OR “Chemotherapeutic Drug*”(Title/Abstract) OR “Antineoplastic Medication*”(Title/Abstract) OR “Anticancer Drug*”(Title/Abstract) OR “Highly Potent Drug*”(Title/Abstract).

- Equation 2 - Medical Informatics Applications.

“Medical Informatics Applications”(Mesh) OR “Medical Informatics Application*”(Title/Abstract) OR “Online System*”(Title/Abstract) OR “Clinical Informatic*”(Title/Abstract) OR “Health Informatic*”(Title/Abstract) OR “Medical Data Processing”(Title/Abstract) OR “Medical Informatic*”(Title/Abstract) OR “Medical Informatics Computing”(Title/Abstract) OR “Public Health Informatic*”(Title/Abstract) OR “Mobile Applications”(Mesh) OR “Mobile Application*”(Title/Abstract) OR “Mobile App*”(Title/Abstract) OR “Portable Electronic App*”(Title/Abstract) OR “Portable Electronic Application*”(Title/Abstract) OR “Portable Software App*”(Title/Abstract) OR “Portable Software Application*”(Title/Abstract) OR “Tablet Application*”(Title/Abstract) OR “Software”(Mesh) OR “Computer Software”(Title/Abstract) OR “Computer Program*”(Title/Abstract) OR “Software Tool*”(Title/Abstract) OR “Software Engineering*”(Title/Abstract) OR “Computer Applications Software*”(Title/Abstract) OR “Computer Software Application*”(Title/Abstract) OR “Software Design”(Mesh) OR “Software Design”(Title/Abstract).

- Equation 3 - Hospital Pharmacy.

“Pharmacy Service, Hospital”(Mesh) OR “Hospital Pharmacy Service*”(Title/Abstract) OR “Hospital Pharmacy Service*”(Title/Abstract) OR “Hospital Pharmaceutical Service*”(Title/Abstract) OR “Clinical Pharmacy Service*”(Title/Abstract) OR “Hospital Pharmacies”(Title/Abstract) OR “Hospital Pharmacy”(Title/Abstract).

The final search equation was developed for use in the MEDLINE database via PubMed by using Boolean operators to combine the 3 proposed equations (Equation 1 AND Equation 2 AND Equation 3).

This strategy was subsequently adapted to the characteristics of each databases, and the databases were searched from inception to July 30, 2021 (the final equations, in each bibliographic database, can be consulted in Supplementary Annex 1). Additionally, the references lists of the included studies were manually searched to identify additional eligible articles. Furthermore, experts on the current subject were contacted in an attempt to identify gray literature (materials and research produced by organizations outside the traditional commercial or academic publications that are disseminated through other distribution channels).

### 2.5. Final selection processing

For the review and critical analysis, the articles that met the following criteria were chosen:

- Inclusion: relevant to the objectives of the current research; an original article, published in peer-reviewed journals and written in English, Spanish or Portuguese.
- Exclusion: articles for which the full text could not be found, and articles that did not specify a relationship between the intervention and the outcome under study (causality criterion).

Relevant articles were selected by two authors of the present review (SC-B. and JS-V.). To validate the inclusion of the articles, it was established that the interrater agreement (kappa index = IK) should be greater than 0.60 (15). Provided that this condition was met, disagreements would be resolved by consensus among all the authors of the review.

### 2.6. Stages of the HD integral management process

The comprehensive management stages that were taken into account for the comprehensive management of the entire HD process were those included in the work by Bernabeu-Martínez et al. (4). Likewise, from this work the flowcharts of said management were obtained.

### 2.7. Document correction, level of evidence and grade of recommendation

The structural validity of the articles was assessed using the Strengthening the Reporting of OBservational studies in Epidemiology (STROBE) guidelines (16), which contains a list of 22 essential aspects that should be described in each article. For each selected article, one point was assigned for each item present. When an item was composed of several sections, these were evaluated independently, with the same value assigned to each item, and then, an average was calculated (this being the final result of that item) in such a way that in no case exceeded the total score of one point per item.

To determine the level of evidence and its degree of recommendation, the Scottish Intercollegiate Guidelines Network Grading Review Group (SIGN) recommendations (17) were used.

### 2.8. Data extraction

The control of the correction of the data was performed by double tables that allowed the detection of the deviations and their correction through newly consulting the original data.

Duplicate records were excluded using the multiplatform program ZOTERO, a bibliographic reference manager developed by the Center for History and New Media of George Mason University.

To determine the timeliness of the studies, the Burton-Kebler half-period (BK) and the price index (IP) were calculated.

The articles were grouped according to the variables under study to systematize and facilitate the understanding of the results. The following data were extracted: first author, year of publication, country where the work was developed, substance studied, software application used, stages, subject to control, type of intervention performed and main results motivated by the effect of the intervention.

### 2.9. Data analysis

The data obtained from the reviewed studies are presented as frequencies and percentages.

To determine the BK, the median age was calculated according to the time range analyzed, and the PI was calculated by determining the percentage of articles with an age of less than 5 years.

The IK value was used to determine the interrater agreement with respect to eh inclusion of each article. Interrater agreement is considered good when IK > 60% (good or very good agreement).

The scores of the STROBE questionnaire were analyzed using the median, maximum and minimum values. The evolution of this score, in relation to the years of publication, was obtained by Pearson’s correlation analysis.

### 2.10. Ethical aspects

All data were obtained from articles accepted for review. Therefore, and in accordance with Law 14/2007 on biomedical research, the approval of the ethics committee was not necessary due to the use of secondary data.

## 3. Results

When applying the search criteria, a total of 104 references were retrieved, including 41 (38.32%) from MEDLINE (via PubMed), 20 (19.23%) from Embase, SCOPUS and Web of Science, and 1 (0.96%). from the Cochrane Library, LILACS and MEDES. An additional 18 studies were identified by manually searching the references lists of the included articles and by consulting experts.

**Figure 1:**
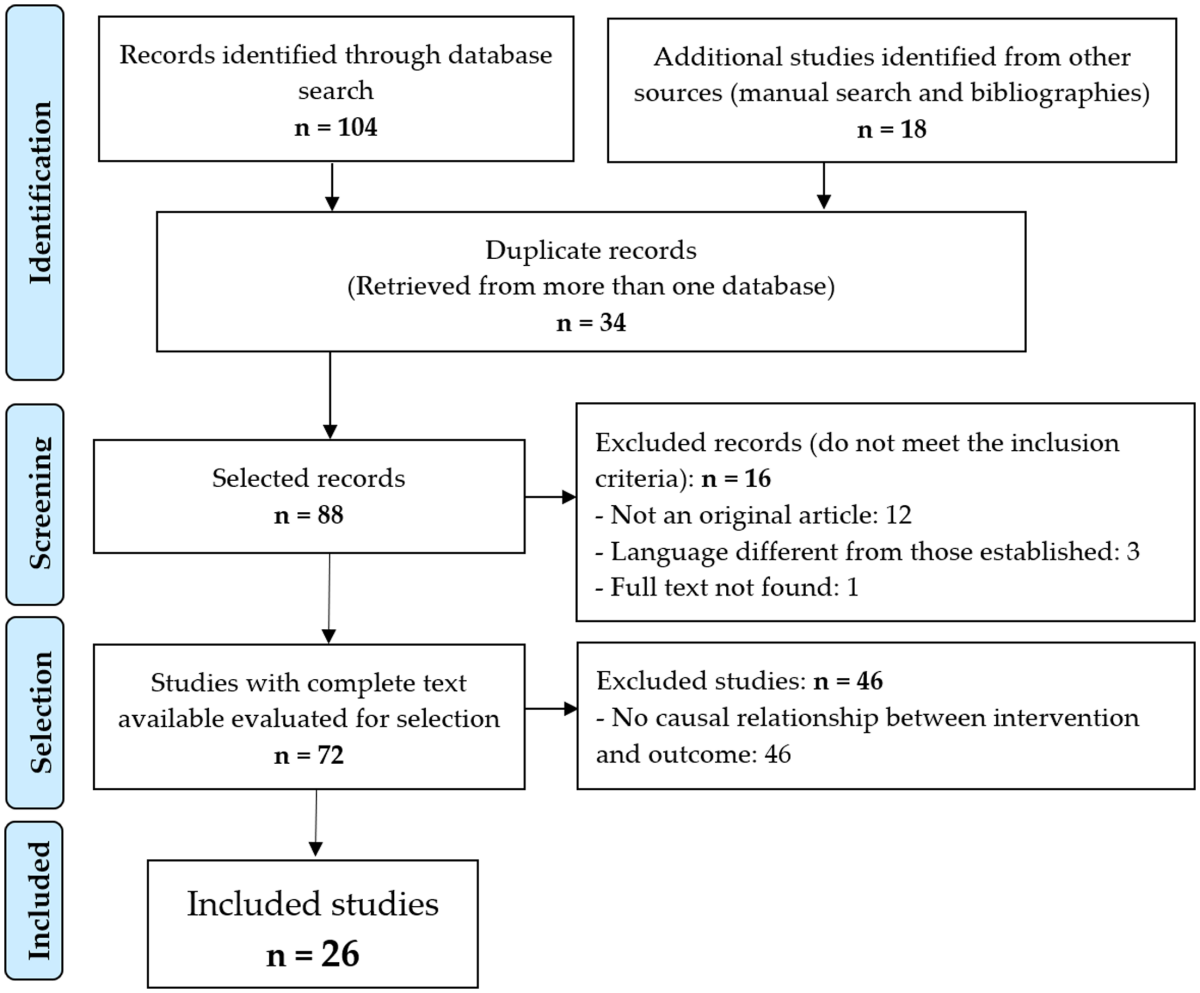
Flowchart of the identification and selection of articles.

After excluding the 34 duplicate records and applying the inclusion and exclusion criteria (Figure 1), 26 original articles (18–43) were included for review and critical analysis (Table 1). The interrater agreement for the selection of the studies among the evaluators was 80.48% (*p* < 0.001).

**Table 1:** Articles selected for the review.

Author/year	Country	Substance	Software application	Controlled stages	Intervention performed	Result
Gayoso-Rey et al. (18)	Spain.	Antineoplastics	Electronic prescription software (Silicon)	Prescription and validation	Consensus to standardize the hospital database of medications and implement the electronic prescription system of an outpatient oncology consultation of a Spanish tertiary hospital	There was a 70% decrease in medication errors.
Terloka et al. (19)	Austria, Czech Republic, Denmark, Germany and Switzerland	Antineoplastics	Workflow software (BD Cato™)	Elaboration	Retrospective analysis of ME (errors in dose volumes) identified during the preparation of antineoplastic drugs in PH services after the introduction of intravenous workflow software with a gravimetric system.	The gravimetric system detected that 7.89% of the 759,060 doses of antineoplastic drugs prepared had errors outside the tolerance range. The proportion of preparations with deviations> 20% ranged from 0.21 to 1.27%, with a mean of 0.71%.
Carrez et al. (20)	Switzerland	Antineoplastics	Workflow software (BD Cato™)	Elaboration	It evaluates the accuracy of manual chemotherapy preparations using three different control methods: no control, double visual verification and gravimetric control with Cato™, in the PH service.	No final preparation (*n* = 438) contained an incorrect drug. The “uncontrolled” method failed to detect 1 of 3 dose errors made and the “double check” failed to detect 3 of 7 dose errors made. The gravimetric control method detected 5 of the 5 dose errors. The precision of the measured doses was equivalent in all control methods (*P* = 0.63). The final preparations were: adequate from 58% to 60%, weakly adequate from 25 to 27%, from 14 to 17% inaccurate and 0.9% incorrect.
Iwamoto et al. (21)	Japan	Antineoplastics	APOTECAchemo robot (AGP^®^ software)	Elaboration	Evaluates the accuracy (safety and feasibility) of the APOTECAchemo robot for the preparation of chemotherapy in a Japanese hospital. The accuracy and safety were compared with the manual preparation in a biological safety cabinet.	Dose accuracy (mean absolute error of the dose) and precision (coefficient of variation) of the robot was 0.83 and 1.04% for the FU and 0.52 and 0.59% for the CPA, respectively. In the manual preparation, these values were 1.20 and 1.46% for FU and 1.70 and 2.20% for CPA, respectively. The absolute dose error of the robotic preparation for CPA was significantly lower than that of the manual preparation (*P* < 0.05).
Ibáñez-García et al. (22)	Spain.	Pharmacotherapy in hospitalized patients (including antineoplastic drugs)	Management software for FH and Oncology (Farhos)	Prescription and validation	It studies all prescriptions issued to adult patients admitted to the hospital for 6 months. All PEs intercepted by pharmacists during the validation process were prospectively audited and entered into a database with the objective of characterizing the severity and potential cost of the PEs that pharmacists can prevent and develop an economic analysis.	A total of 484 PEs were intercepted: 36.2% of PEs were classified as minor severity, 59.1% as moderate and 4.7% as severe. The most common type of moderate-severe PE found was excessive dose (30%, 94/309), followed by insufficient dose (20%, 62/309) and omission (19%, 58/309). One of the most frequent families of drugs involved in moderate-severe PD were antineoplastic drugs (22.3%, 69/309). The probability of suffering an adverse drug event (PAE) was greater than 40% in 49% of the PEs.
Pacheco Ramos et al. (23)	Spain.	Antineoplastics	APOTECAchemo robot (AGP^®^ software)	Elaboration	Describes the implantation of a robot for the preparation of antineoplastics in the FH service and evaluates the added value to the pharmacotherapeutic process (robot performance and gravimetric control)	Dosing errors were identified and avoided in 1.12% (*n* = 133) of the preparations, which did not affect the patient when identified by the robot.
Mattsson et al. (24)	Denmark	Antineoplastics	Electronic prescription software (local/in-house development)	Prescription and validation	Determines the incidence, type, severity and related risk factors for prescription dose errors not intercepted in two medical oncology centers. One center used CPOE and the other center used paper prescriptions. Subsequently, all prescriptions were reviewed and the prescribed doses were recalculated according to the guidelines of each center.	They evaluated a total of 5,767 prescriptions for 2.5 months, 2,677 from the hospital with CPOE and 3,090 from the hospital with paper prescription. The crude analysis shows a general risk of a prescription dose error of 1.73 per 100 prescriptions. The CPOE resulted in 1.60 and the paper prescription forms in 1.84 errors per 100 prescriptions, that is, OR = 0.87 (95% CI: 0.59–1.29, *P* = 0.49).
Bedouch et al. (25)	France	Pharmacotherapy in hospitalized patients (including antineoplastic drugs)	Act-IP © website	Documentation	They analyze the IP documented in Act-IP© over a period of 30 months.	A total of 34,522 PIs were registered by 201 pharmacists working in 59 hospitals. PIs were mainly related to “dose adjustment” (25%), “drug interruption” (20%) and “drug change” (19%). The acceptance of physicians was significantly associated with the therapeutic group: antineoplastics and immunomodulators [OR = 2.29, 95% CI (1.94–2.69)].
Bazin et al. (26)	France	Antineoplastics	Multispec^®^ Analyzer (integrated software)	Elaboration	Evaluates the qualitative calibration by the percentage of correct recognition of the MP, and the quantitative calibration by the percentage of correct concentration, in the routine control of a centralized chemotherapy unit in a university hospital after 24 months.	A total of 23,350 preparations were routinely analyzed, 4% (*n* = 936) were rejected/invalidated by the first analysis and another sampling was requested after homogenization. 850 of these preparations finally passed a second analysis. In total, 86 preparations had to be produced again (0.37%). Regarding the main errors observed, 77 preparations were rejected due to a gap greater than 15% between the theoretical and calculated concentrations. Only 3 samples were discarded due to a mismatch of molecules. Three errors were also observed in the nature of the vehicle and 3 in the volume of the vehicle. Regarding the qualitative results, the recognition of the analyzed samples was correct in terms of molecules for 97 and 99.95% in terms of vehicle. In general, a lack of homogeneity has been observed between samples containing the same drug, both in terms of recognition and in terms of quantification.
Adelson et al. (27)	United States	Antineoplastics	Electronic prescription software (Epic Beacon)	Prescription	Describes the implementation of electronic prescription for chemotherapy. To monitor the use of evidence-based treatments, a new quality measure is created: the REBA.	The overall REBA of 0.86 significantly exceeded the prespecified target of 0.80 (*P* = 0.001). The REBA ranged from 0.50 to 0.95 between the disease groups. The use of antiemetics increased by 20% after the implementation of Beacon. User satisfaction at 8 months ranged from 76 to 80%.
Meisenberg et al. (28)	United States	Antineoplastics	Electronic prescription software (Epic Beacon)	Prescription	Evaluates the amount and type of errors associated with three different methods of chemotherapy prescription used sequentially: handwritten prescriptions, preprinted prescriptions and CPOE.	The rate of problematic order sets, those that require a significant revision for clarification, decreased from 30.6% with handwritten prescriptions to 12.6% with preprinted prescriptions (preprinted versus manuscripts, *P* < 0.001) to 2. 2% with CPOE (preprinted vs. CPOE, *P* < 0.001). The incidence of errors capable of causing harm was reduced from 4.2% with handwritten orders to 1.5% with preprinted orders (preprinted versus handwritten, *P* < 0.001) to 0.1% with CPOE (CPOE v preprinted, *P* < 0.001).
Aita et al. (29)	Italy	Antineoplastics	Electronic prescription software for chemotherapy (G2 system) (local/in-house development)	Prescription	It evaluates the frequency, type, prevention capacity, as well as the potential and actual severity of outpatient chemotherapy prescription errors in an Oncology Department where CPOE is used, during a period of 24 months. Severity was defined according to the severity scale of the Mode of Effect Analysis and Health Care Failures.	The overall error rate was 20%. If systematic errors (that is, errors due to an initially defective implementation of chemotherapy protocols in computerized dictionaries) are excluded from the analysis, the error rate was reduced to 8%. Incomplete prescriptions were the most frequent (66%), followed by incorrect and inappropriate prescriptions (28% and 6%, respectively). Most of the errors were considered definitely avoidable. According to the presumed potential damage error, 72% were classified as minor; only 3% had the potential to produce serious or catastrophic injuries. 68% were classified as near misses; Adverse drug events had little or no effect on clinical outcome.
Chen et al. (30)	Taiwan	Antineoplastics	CytoCare robot (CytoPlan software)	Elaboration	Two-month evaluation of the performance of the robot in terms of its success rate and summarizes the causes of failure, and if the robot can reduce the FTE of the oncology pharmacy.	The total number of doses performed was 1,028: 123 doses (12.0%) failed. The causes of failure were classified into two groups: aerial (98 of 1,028 total doses, 9.53%) and nonaerial (load: 7 in 1,028 doses, 0.68%; restraint: 7 in 1,028 doses, 0.68%; trash: 6 in 1,028 doses, 0.58%; and others), all improved after the change of syringe systems and improvements introduced by the engineers.
Elsaid et al. (31)	United States	Antineoplastics	Electronic prescription software for chemotherapy (Adobe Acrobat)	Prescription	Evaluates the impact of the implementation of CPOE for chemotherapy with standardized templates on the incidence and types of prescription errors. Standardized and specific chemotherapy prescription forms are developed and implemented.	The monthly error rate prior to implementation remained stable with 16.7 errors prevented per 1,000 doses of chemotherapy. A 30% reduction in prescription errors was observed at the beginning of implementation. After implementation, a negative change in the slope of prescription errors was observed (coefficient = −0.338; 95% CI: −0.612 to −0.064). The estimated RR of transcription errors was 0.74; 95% CI (0.59–0.92). The estimated RR of dose calculation errors was 0.06; 95% CI (0.03–0.10). The estimated RR of frequency/duration errors of chemotherapy was 0.51; 95% CI (0.42–0.62).
Seger et al. (32)	United States	Antineoplastics	CytoCare robot (CytoPlan software)	Elaboration	Evaluates the impact of a robotic device that prepares antineoplastic and adjuvant drugs on patient and staff safety, the accuracy of the medication determined by gravimetric techniques, the efficiency of the workflow, compared to the preparation by manual methods.	A total of 1,421 doses were prepared manually and 972 were prepared by robot, found 9 (0.7%) and 7 (0.7%) serious medication errors (*P* = 0.8) and 73 (5.1%) and 28 (2, 9%) personnel security events (*P* = 0.007), respectively. A total of 12.5% (23 of 184) of the manual preparations and 0.9% (one of 110) robot preparations were not adequate (*P* < 0.001).
Collins and Elsaid (33)	United States	Antineoplastics	Electronic prescription software for oral chemotherapy (Siemens Invision CPOE system)	Prescription	An HFMEA is developed on the oral chemotherapy process and the implementation of the CPOE of oral chemotherapy validated by the pharmacist of the PH service in a tertiary hospital with an oncology day hospital area is evaluated. The incidence of prescription errors is compared before and after the implementation of electronic prescribing.	The HFMEA hazard analysis revealed seven possible failure modes, with the highest hazard scores in the prescription and process management components. The implementation of electronic prescribing significantly reduced (*P* = 0.023) the risk of prescription error by 69% and eliminated certain types of errors that can lead to significant harm to the patient.
Harshberger et al. (34)	United States	Antineoplastics	Electronic medical record software (Epic, Verona, WI) and electronic prescription for chemotherapy (Epic Beacon)	Prescription and Documentation	Evaluates the implementation of an EHR system with CPOE for outpatient chemotherapy in an outpatient cancer center in a hospital. Three outcome measures are evaluated: overall completeness score by type of chart, completeness score by regimen, and staff satisfaction with EHR/CPOE versus paper charts.	A total of 90 patient records (45 EHR/CPOE tables and 45 paper tables with matching regimens) were randomly selected and reviewed. The overall integrity of the documentation was 93% for EHR/CPOE compared to 67% for paper charts (*P* = 0.001). The EHR/CPOE system improved the documentation with respect to the paper tables for the following elements: MRN, cycle duration, height/weight/BSA, dose per unit, diluent, duration of infusion, previous and subsequent medication, results of laboratory, treatment parameters, pharmacy interventions, follow-up plan and clinical trial notes.
Chen and Lehmann (35)	United States	Antineoplastics	Electronic prescription software for chemotherapy (The Eclipsys/Allscripts Sunrise system)	Prescription	Implements the CPOE system of the hospital in the area of pediatric oncology.	The proportion of chemotherapy prescriptions sent using a specific research protocol or sets of standard care orders increased from 57 to 84% as the number of active order sets increased to 200. The number of related patient safety events with medication decreased 39% after the implementation of CPOE in pediatric oncology.
Nerich et al. (36)	France	Antineoplastics	Electronic prescription software for chemotherapy (BPC^®^)	Prescription	Prospectively analyzes all consecutive orders of prescription drugs during 1 year, to determine the incidence of PE and to analyze the PE related to antineoplastic treatment in teaching hospitals with CPOE.	The incidence of PME errors was estimated at 1.5% (1.3–1.7), with a significant or very significant potential clinical impact in 62.9% of cases. Life-threatening events were avoided in 3.7% of cases. In general, the incidence of PME related to a significant, very significant or vital potential clinical impact was estimated at 1% (0.8–1.2). The most common type of error was related to the dosage of the antineoplastic (61%). More than 20% of PMEs are medication errors directly related to medication prescription.
Murphy (37)	Canada	Antineoplastics	Electronic prescription and dispensing software for chemotherapy	Prescription and dispensing	It compares a web-based capecitabine prescription and dispensing software with traditional manual methods with the participation of oncologists and pharmacists.	The total time of the task was reduced with the use of the software by 33% for oncologists and 26% for pharmacists. The use of resources by oncologists when using the software went from an average of 8.27 resources (range of 6–10) with 14 used in general to an average of 5.43 (range of 5–6) with 9 used in total, while for pharmacists it went from an average of 8.4 resources (range of 6–12) with 12 used in general to an average of 10.5 (range of 6–12).
Small et al. (38)	United Kingdom	Antineoplastics	Electronic prescription software for chemotherapy (VARIS MedOnc)	Prescription	It evaluates the prescription errors detected with CPOE versus prescriptions through spreadsheets in patients with outpatient chemotherapy, through a prospective audit of 8 months.	The CPOE reduced errors by 42% (RR 0.58; 95% CI: 0.47–0.72). Errors occurred in 20% of the spreadsheet recipes compared to 12% of the computerized recipes. The proportion of errors that were minor decreased and that of serious errors increased with little change in the proportion of significant or life-threatening errors.
Voeffray et al. (39)	Switzerland	Antineoplastics	Electronic prescription software for chemotherapy (File Maker Pro)	Prescription	Evaluates the effect of the CPOE on the prescription errors measured in the periods of 15 months before and 21 months after starting with the CPOE. Errors were classified into major (dose and name of the drug) and minor (volume or type of infusion solution).	Before computerization, 141 errors were recorded for 940 prescribed chemotherapy regimens (15%). After the introduction of the CPOE system, 75 errors were recorded for 1,505 prescribed chemotherapy regimens (5%). Of these errors, 69 (92%) were recorded in recipes that did not use a computerized protocol.
Huertas Fernández et al. (40)	Spain.	Antineoplastics	Electronic prescription software for chemotherapy (Oncowin version 4.0)	Prescription	Evaluates the impact of chemotherapy CPOE on the reduction of ME.	At least one error was detected in 100% of the manual prescriptions (*n* = 30) and in 13% of the electronic prescriptions (*n* = 30) (*P* < 0.001). The median of errors by CPOE was 0 (range: 0-1), while in the manual prescriptions the median was 5 (range: 1–12) (*p* < 0.001). The CPOE and the subsequent validation in the PH service have helped reduce dosage errors from 10% to 3.3%.
Kim et al. (41)	United States	Antineoplastics	Electronic prescription software for chemotherapy (RxTFC Pharmacy Information System)	Prescription	Implements and evaluates the impact of CPOE in the reduction of errors in pediatric chemotherapy orders.	After the implementation of CPOE, daily chemotherapy orders were less likely to have an inadequate dosage (RR, 0.26; 95% CI, 0.11–0.61), of incorrect dosage calculations (RR, 0.09; 95% CI, 0.03–0.34), missing cumulative dose calculations (RR, 0.32; 95% CI, 0.14-0.77) and incomplete nursing checklists (RR, 0, 51; 95% CI, 0.33–0.80). There was no difference in the probability of an inadequate dosage.
Krampera et al. (42)	Italy	Antineoplastics	Electronic prescription, administration and pharmaceutical validation software (GRC, “Gestione Relazioni con il Cittadino,” made by Accenture™)	Prescription, validation and administration	Evaluates CPOE in a bone marrow transplant unit.	Despite the large number of procedures, the computerized system effectively replaced the oral and handwritten transmission of information between medical personnel, pharmacists and nurses, and reduced the risks of error. In addition, it contributed to the medical update through warnings about possible problems in case of multiple drug prescriptions, and provided the PH service with a valuable tool to monitor the use of medications.
Beer et al. (43)	Canada	Antineoplastics	Electronic prescription software for chemotherapy (integrated in the electronic medical record: OpTx)	Prescription	It evaluates the average time needed to review the electronic prescriptions in comparison with the existing paper method, and to determine if the CPOE would decrease the IP rates in two of the main tertiary centers of the ACB during a month.	Among the 836 chemotherapy orders reviewed, the average review time of the pharmacist orders increased by 5.15 min with the implementation of an electronic order entry system. A total of 62 PIs were recorded in the study. The PI rate was 7.14% for electronic orders and 7.47% for manual paper orders.

PH, hospital pharmacy; FU, fluorouracil; 5-FU, 5-fluorouracil; CPA, cyclophosphamide; PE, prescription errors; PAE, probability of suffering an adverse drug event; CPOE, computerized physician order entry; PI, pharmaceutical intervention; MP, dangerous drug; REBA, evidence-based adherence rate; FTE, full-time equivalents; HFMEA, Healthcare Failure Mode and Effects Analysis; ACB, Alberta Cancer Board; EHR, electronic medical records; MRN, medical record number; BSA, body-surface area; PME, medication prescription errors; ME, medication errors; CI, confidence interval; OR, Odds ratio; RR, relative risk.

The selected articles presented an obsolescence, according to the BK semiperiod (8 years), with a PI of 15.4%.

When evaluating the document correction of the included articles through the STROBE questionnaire, the scores ranged from 6.5 (32.5% compliance) to 19.1 (95.5% compliance), with a median of 14.3 (see Table 2). There was a weak, nonsignificant linear trend over time (*R*^2^ = 7.1; *p* = 0.187).

**Table 2:** Analysis of the documentary quality of the studies through the 22 assessment items of the STROBE guide.

Article	Score of the items of the questionnaire																							
	1	2	3	4	5	6	7	8	9	10	11	12	13	14	15	16	17	18	19	20	21	22	Total	%^b^
Gayoso-Rey et al. (18)	1	1	1	1	0	1	0	0	0	NA^a^	1	0	0.3	0.5	1	NA	0	0	1	1	1	1	11.80	59.00
Terloka et al. (19)	1	1	1	0	0	1	1	1	0	NA	1	0	0	0	1	NA	1	1	1	1	1	1	14.50	72.50
Carrez et al. (20)	1	1	1	1	0	1	1	1	0	NA	1	1	0.6	0.5	1	NA	1	1	1	1	1	1	16.60	83.00
Iwamoto et al. (21)	0.5	1	1	0	0	1	1	1	0	NA	1	1	0	1	1	NA	0	1	0	1	1	1	13.80	69.00
Ibáñez-Garcia et al. (22)	1	1	1	1	1	1	1	1	1	NA	1	1	0.6	0.5	1	NA	1	1	1	1	1	1	19.10	95.50
Pacheco Ramos et al. (23)	0.5	1	1	0	1	1	1	1	0	NA	1	0	0.6	1	1	NA	1	0	0	1	1	1	14.10	70.50
Mattsson et al. (24)	1	1	1	1	1	1	1	0	0	NA	1	1	0.3	0.5	1	NA	1	1	1	1	1	1	16.80	84.00
Bedouch et al. (25)	0.5	1	1	1	0	1	1	1	1	NA	1	1	0.6	0.5	1	NA	1	1	1	1	1	1	17.60	88.00
Bazin et al. (26)	0.5	1	1	0	0	1	1	1	1	NA	1	0	0	0	1	NA	0	1	0	1	1	1	13.30	66.50
Adelson et al. (27)	0.5	1	1	0	0	0	1	0	1	NA	1	0	0.3	0	1	NA	0	0	0	1	1	1	9.80	49.00
Meisenberg et al. (28)	0.5	1	1	0	1	0	1	1	0	NA	1	1	0.3	0.5	1	NA	1	1	1	1	1	1	15.30	76.50
Aita et al. (29)	1	1	1	1	1	1	1	1	1	NA	0	0	0.6	0.5	1	NA	1	1	1	1	1	1	17.10	85.50
Chen and Lehmann (30)	0.5	1	1	0	0	1	1	1	0	NA	1	0	0.3	0.5	1	NA	1	0	0	1	0	0	10.30	51.50
Elsaid et al. (31)	1	1	1	0	1	1	1	1	0	NA	1	1	0.3	0.5	1	NA	1	1	0	1	1	1	15.80	79.00
Seger et al. (32)	1	1	1	0	1	0	1	1	1	NA	1	1	0.3	0.5	1	NA	1	1	1	1	1	1	16.80	84.00
Collins and Elsaid (33)	1	1	1	0	1	1	1	1	0	NA	0	1	0.3	0	1	NA	1	0	1	1	1	0	13.30	66.50
Harshberger et al. (34)	1	1	1	1	1	1	1	1	1	NA	1	1	0	0.5	1	NA	0	1	0	1	1	1	16.50	82.50
Chen and Lehmann (35)	0.5	1	1	0	0	0	0	0	0	NA	0	0	0	0	1	NA	0	0	0	1	1	1	6.50	32.50
Nerich et al. (36)	1	1	1	0	1	1	1	1	0	NA	1	1	0.3	0.5	1	NA	1	1	1	1	1	0	15.80	79.00
Murphy (37)	0.5	1	1	0	0	1	0	1	0	NA	1	0	NA	0.5	1	NA	1	1	0	1	1	0	11.00	57.89
Small et al. (38)	1	1	1	0	0	1	1	1	1	NA	1	1	0.3	0	1	NA	1	1	1	1	1	0	15.30	76.50
Voeffray et al. (39)	0.5	1	1	0	0	0	1	1	0	NA	1	0.3	0.5	1	1	NA	0	1	1	1	1	0	12.30	61.50
Huertas Fernández et al. (40)	1	1	1	0	1	1	1	1	0	NA	1	0.3	0	1	1	NA	0	1	1	1	1	0	13.30	66.50
Kim et al. (41)	1	1	1	0	1	1	1	1	0	NA	1	0	0.3	0	1	NA	0	0	1	1	1	1	13.30	66.50
Krampera et al. (42)	0.5	1	1	0	1	0	0	0	0	NA	0	0	0.3	0.5	0	NA	0	1	0	1	1	0	7.30	36.50
Beer et al. (43)	0.5	1	1	0	1	1	1	1	1	NA	1	1	0.6	0	1	NA	1	1	1	1	1	0	15.60	78.00

^a^NA = not applicable.

^b^Percentage of compliance with all items, excluding those that do not apply (NA).

According to the SIGN criteria, this level of evidence among the included studies was 2 ++ (systematic reviews with a high probability that the relationship is causal) with recommendation grade of B (a body of evidence that includes studies directly applicable to the target population and that demonstrate overall consistency of the results).

Eight studies were published in English (27, 28, 31–35, 41), and 4 studies were published in Spanish (18, 22, 23, 40).

### 3.1. Controlled hazardous substance

All studies examined the management of antineoplastic drugs. Two studies (22, 25) were focused on the management of pharmacotherapy among hospitalized patients, although they also included antineoplastic drugs.

### 3.2. Computer application used

Seventeen studies described computer software related to electronic prescription (18, 24, 27–29, 31, 33–43). The article by Murphy (37) also described software for managing and dispersing drugs, and the article by Krampera et al. (42) described the administration and validation of drugs. In addition, Harshberger et al. (34) described the use of electronic medical records software.

Terloka et al. (19) and Carrez et al. (20) described workflow software, and Ibáñez-Garcia et al. (22) described a program for the managing antineoplastic drugs in hospital pharmacies.

Bedouch et al. (25) described a website, and 4 other studies described the use of robots for the development of HDs: 2 APOTECAchemo robots (AGP^®^ software) (21, 23) and 2 CytoCare robots (CytoPlan software) (30, 32). Finally, 1 study described a Multispec^®^ analyzer (integrated software) (26).

Stages of the controlled hazardous substance management process.

Among the reviewed studies, none reported using a standardized system that ensured the integral management of HD (see Table 3).

**Table 3:** Stages controlled by the different articles reviewed.

Controlled stage	Frequency (%)	Article reference
Prescription	12 (46,20)	(27–29, 31, 33, 35, 36, 38–41, 43)
Prescription and validation	3 (11,50)	(18, 22, 24)
Elaboration	7 (26,90)	(19–21, 23, 26, 30, 32)
Documentation	1 (3,80)	(25)
Prescription and documentation	1 (3,80)	(34)
Prescription and dispensing	1 (3,80)	(37)
Prescription, validation and administration	1 (3,80)	(42)

### 3.3. Main interventions carried out

Of the 19 interventions related to electronic prescriptions (18, 22, 24, 25, 27–29, 31, 33–43), six studies described the implementation of an electronic prescription system (18, 27, 31, 34, 35, 41).

The most commonly reported results were the incidence and/or type of electronic prescription error (22, 24, 25, 28, 29, 31, 33, 35, 36, 38–42). Six articles compared these data with previous methods, i.e., paper prescription (24, 28, 39–42), preprinted prescriptions (28) and spreadsheets (38).

Other outcomes included the severity (22, 24, 29), the associated risk factors (24, 26), the cost (22) and the ability to prevent prescription errors (29). Pharmaceutical interventions were also analyzed with respect to the process of validating prescribed treatments (22, 25); the evidence-based adherence rate (REBA) (compliance with clinical guidelines) (26); the correct completion of medical history, clinical and electronic prescription (34); and the time and resources used in the process of prescribing and validating oncological treatments (37, 43).

In two studies, an analysis of “mode of effect and failures” was conducted to define the severity of prescription errors (29) and examine the process of oral chemotherapy (33). In two others, a survey of staff satisfaction with electronic prescription versus paper prescription was conducted (27, 34).

All investigations were carried out in oncological inpatients and/or outpatients, except for 2 studies that also included nononcological patients (22, 25), 1 study that included pediatric oncological patients (40) and 1 study that focused on bone marrow transplantation patients (42).

The 7 interventions performed in the development stage focused on quantitatively evaluating the accuracy of the chemotherapy preparations (19–21, 23, 26, 30, 32) when incorporating the software. Among these interventions, 3 compared the software with the previous methods, i.e., manual preparations without control or double visual verification versus gravimetric control (20) and manual preparations versus preparation robot (21, 32). Only one study qualitatively evaluated chemotherapy preparations (26).

Finally, 3 interventions evaluated the performance of a robot (23, 30, 32); one in one of these studies, the robots also prepared the adjuvant medication (32).

**Figure 2:**
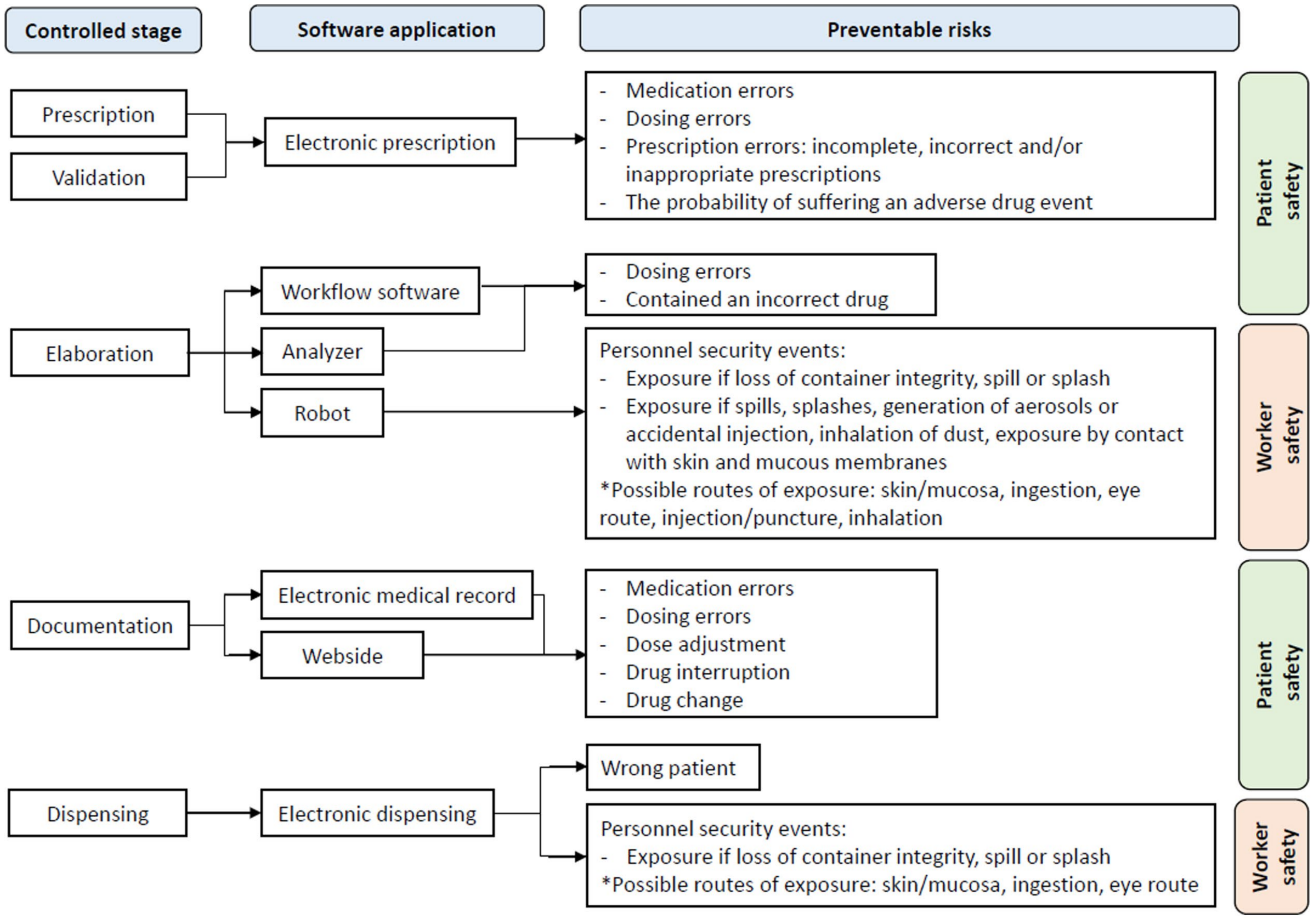
Preventable risks at each stage of HD management.

Figure 2 visually proposes the risks that should be prevented in each of the stages that have been studied in Table 3.

### 3.4. Results obtained as a cause of the intervention

The decrease in medication errors due to electronic prescribing (the most common intervention) was highlighted in most studies (18, 24, 28, 29, 31, 33, 35, 38, 40, 42), association in 5 (28, 31, 33, 38, 41).

The most common type of prescription error was dosage error (22, 24, 25, 29, 31, 36, 40, 41), followed by a lack of integrity in the prescriptions (29, 34). The majority of prescription errors were classified as minor or mild (22, 29); most moderate-to-severe errors were dosage errors (22).

The studies that evaluated the time it takes for the prescription and its validation (37, 43) showed that the use of the software reduced the task time by 33% for oncologists and 26% for pharmacists (37). On the other hand, one study observed that task time increased by 5.15 min (43).

A greater number of dosing errors were detected due to the implementation of gravimetric systems (19, 20), a processing robot (23) and a sample analyzer (26). Dosing errors were reduced with the use of processing robots compared to manual preparations, with this statistically significant difference (21, 32).

The only study that measured personnel safety events observed a reduction in such events (32); two studies assessed staff satisfaction with electronic prescribing (27, 34).

## 4. Discussion

According to the recommendations on the objectives of a systematic review (44), the current review synthesized information related to interventions for managing HDs in hospital pharmacy services and the software used in the management process. This review aimed to provide relevant information to the scientific community to promote new interventions for worker protection. The results indicate that although software is used for the prescription and preparation of HDs – mainly antineoplastic drugs –no study described a software that controlled the integral management of HDs within the hospital pharmacy services.

The development of new technologies, especially derived from Web 2.0, has led the information and communication industries to play an increasingly important role in a global economy. For this reason, there are studies that conclude that the future development of this industry must take into account the privacy and control of the personal data of its patients and users (45).

Thus, other studies have concluded that mHealth apps could be used to predict the behavior of patients in the face of preventive recommendations and to monitor the symptoms of users in home care (46).

The obsolescence of the articles reviewed was similar to that found in a previous systematic review related to occupational health and exposure to HDs (2, 47). This is confirmed by the low percentage of articles included herein that were published within the past 5 years, thus indicating the need for updated findings.

The evaluation of the document correction of the studies included in this review was performed using STROBE and did not reveal any temporal evolution. Typically, the most recent articles present better results and are linked to the progressive implementation of quality questionnaires. In fact, the oldest studies did not usually follow these quality guidelines; for example, the first documents on STROBE date from 2004, and their use was gradual (48). It should be noted that in the vast majority of the included studies, all the measures adopted to address potential sources of bias were not specified, nor were additional interaction of sensitivity analyses performed. All this is the consequence of not having obtained higher scores.

The level of evidence and grade of recommendation that this study would obtain according to the SIGN criteria are consistent with those observed in previous studies (49). Despite seeking a consistent cause-effect relationship, since intervention studies were examined, this circumstance was not always fulfilled. Some types of studies are more prone to bias than others (49). It is known that many studies of occupational health and safety are still not based on the highest-quality evidence (50). This may be due to the limitation of certain designs of primary studies, such as clinical trials, which are considered robust but may not be adequate to evaluate interventions in occupational health because they generally present very long-term effects. In this review, the management and control of HDs are not the most studied causes.

The predominance of American studies in reviews is widely observed in the scientific literature. The power of the country’s universities and the significant public and private funding of its institutions and research centers contribute to this. Furthermore, most of the included studies were published in English because this language is predominant in health science publications (51). Moreover, the number of English-language journals contained in the main bibliographic databases is very high, and publishing in these journals increases an article’s chances of citation (52).

Almost all computer applications described in the reviewed studies were dedicated to controlling antineoplastic drugs, a logical situation since they are drugs classified as high risk, and their incorrect use presents a greater probability of causing serious or even fatal damage to patients (53). Antineoplastic drugs are the second most common cause of death due to medication errors (54), so they are a priority in all clinical safety programs that are established in hospitals to improve the safety of drug use. The U.S. National Quality Forum included the “improvement of the safety of high-risk drugs” among the 30 fundamental safety practices for widespread implementation in all hospitals (55).

Similarly, it is important to consider the high complexity of high-risk drugs when they are used in hospitals, so it is necessary to intervene in each and every one of their stages, highlighting packaging, storage, prescription, validation, preparation, and dispensing. Administration and disposal, with many of these stages being the responsibility of the hospital pharmacy service (56).

Therefore, it is essential to carry out specific practices that avoid errors and do not cause adverse effects in patients. For this, and thanks to the development of new technologies, programs and tools have been designed and implemented to improve drug safety and reduce possible errors caused by the human factor (57). It should be noted that two meta-analyses found that electronic prescribing reduced medication-related errors by half (58, 59).

In the present review, the most commonly used computer application was the one for electronic prescription and, second, those for preparation. This result was predictable since it has long been known that the complexity of antineoplastic treatments means that most medication errors are found in the stages of prescription (60), administration and preparation (61, 62). These errors (together with their toxicity associated with treatment) may have a severe impact on patients. If, in addition, it is taken into account that medication errors are preventable in most cases (63, 64), it is normal that technological solutions have focused on these stages, reducing their complexity, such as standardization and simplification of protocols and procedures, automation of calculations (drug dose according to weight, body surface, renal function, among others), incorporation of restrictions that prevent unauthorized processes (implementing multiple alarms in the prescription: variation of dose between two cycles, excess of maximum and accumulated dose, improbable interval between two prescriptions, etc.) and decreasing the possibility of human error (gravimetric quality control, processing and dispensing robots, etc.). These solutions have led to a significant reduction in medication-related errors.

This predominance of publications focused on prescription software is consistent with the general trend of efforts and resources focused on patient safety with mandatory quality standards and rather than worker safety, possibly due to a lack of worker safety regulations (2).

Other computer applications examined in this review were those involved in the development, specifically in the quantitative and qualitative quality control of antineoplastic preparations intended for administration; these applications also aimed to increase patient safety. Here, we highlight the difficult challenge of designing an accurate and efficient preparation method that also boasts a high level of safety with the aim of preventing HD exposure among health workers. The use of robots in drug preparation is a partial solution to the manual preparation, as it helps to avoid or reduce HD exposure (in the form of spills, exposure to aerosols and needle sticks) as well as reduce the stress of preparation, human error and lack of traceability of the preparation.

Health workers are exposed to the toxic effects of these HDs while preparing or administering chemotherapy (9). There are no equal measures for their protection or for the protection of patients, since the latter assume their risk of exposure to HDs to cure their disease, while it is not necessary for workers to assume that risk.

While the use of automation technology is increasing, it is not being implemented at the rate that would be needed since most hospitals continue to work with manual preparation and dispensing methods. There are several reasons for this delay, including the high price of this technology and the lack of strict standards for employee safety and environmental control. However, there is legislation that supports the safety of the environment (65).

It would not be appropriate to end this section without recognizing that, currently, there are other management applications of some parts of the HD process that have not appeared in the review or that have not been evaluated in the scientific literature.

Regarding the stages of the HD management process, as already mentioned, in the reviewed studies, no software was found that would perform the integral management of the entire HD process controlling all its stages, and in addition, there is no traceability. of the processes or minimization of the risks associated with these drugs.

At this point, attention was drawn to the lack of publications related to the rest of the stages of HDs other than prescription, which are also very important in the process of managing these drugs, both due to their complexity and risk of exposure as well as their costs (including materials, equipment and labor), such as the preparation, dispensing and disposal of HDs waste, and that their responsibility falls mainly on hospital pharmacy services and their workers. In addition, these steps carry risks both for patients (errors in preparation, dispensing, etc.) and for workers (mainly exposure to HD).

Most of the interventions related to electronic prescribing published results of incidence of prescription errors (dosing error predominant), showing, in all cases, a decrease after the implementation of electronic prescribing, giving strength to this safety measure.

Regarding the preparation stage, articles that used a processing robot for the preparation of HDs were predominant, followed by gravimetric control software. All their observed results showed that the technology improved the accuracy of the preparations, so the widespread implementation of these software is essential to continue improving the quality of the preparations.

### 4.1. Critical analysis of the authors

It was noteworthy that in the studies on the development of HDs wherein a robot was used, the risk of exposure among workers was not evaluated. This was especially true when this circumstance was one of the arguments for the implementation of robots. Compulsory quality standards are available for the preparation of certain drugs, but there are no specific standards for the prevention of HD exposure among health workers who preparing or administering these drugs.

Consistent with the findings of Bernabeu-Martínez et al. (4), dangerous drugs should be integrated into a standardized management system to improve the safety of the patients and health professionals while enhancing the efficiency of resources and reducing the risk of the processes, thereby guaranteeing the quality and safety of the HD management process. Carrying out a risk assessment in accordance with a systematic methodology and using a preventive approach would allow us to calibrate the probability of occurrence and the severity of any adverse event.

Only 2 studies examined the level of satisfaction with the implementation of the computer application among workers; specifically, these studies examined satisfaction with the use of electronic prescription. The current authors consider it important and necessary to measure worker satisfaction, despite that fact that most of the selected studies neglected to do so.

### 4.2. Limitations

Although systematic reviews should be based on studies with follow-ups and studies with a high level of scientific rigor, all relevant studies were included in this analysis. The results of the present review are limited by the shortcomings of each included study. Furthermore, as in many other studies related to occupational health, it is nearly impossible to included only studies with high-level designs and high levels of evidence (51, 66).

The high rate of nonrelevant articles retrieved compared to the number of articles that were ultimately included (28) can be considered a possible limitation of this review. It is important to consider that the Scopus and Web of Science databases yielded documents that were ultimately irrelevant; this phenomenon could be due to the lack of indexing (the search was performed using text to search the title, abstract and keywords). This high level of “documentary noise” has been observed in previous systematic reviews (2, 67). In addition, although an exhaustive search was conducted, it is possible that some relevant studies were not identified.

## 5. Conclusion

Among all the software developed for the management of HDs, the electronic prescription software for antineoplastic drugs was the most widely implemented software at the hospital level.

No software was found to control the entire HD process.

Only one of the selected studies measured safety events in workers who handle HDs. Health professionals reported being satisfied with the implementation of this type of technology for their daily work with HDs.

All studies reviewed herein considered patient safety as their final objective. However, none of the studies evaluated the risk of exposure to HDs among workers.

For all these reasons, in line with the work of Bernabeu-Martínez et al. (2), it would be convenient to apply information and communication technologies to manage the processes that involve HD in a more complete and simple way and avoid the associated risks.

## Author contributions

JS-V and PG-S supervised and carried out the conception of the work. SC-B and JS-V designed the study, collected the data, and prepared the database. SC-B, PG-S, and JS-V analyzed, interpreted the data, and edited the first draft. All authors participated equally in the critical review and editing of the article, read and agreed to the published version of the manuscript, and contributed substantially to the present study.

## Funding

Partial financial support for translation and publication was received from the Alicante Biomedical and Health Research Institute (ISABIAL).

## Conflict of interest

The authors declare that the research was conducted in the absence of any commercial or financial relationships that could be construed as a potential conflict of interest.

## Publisher’s note

All claims expressed in this article are solely those of the authors and do not necessarily represent those of their affiliated organizations, or those of the publisher, the editors and the reviewers. Any product that may be evaluated in this article, or claim that may be made by its manufacturer, is not guaranteed or endorsed by the publisher.

## Abbreviations

HDs: dangerous drugs
LILACS: Latin American and Caribbean Literature in Health Sciences
MEDES: Medicine in Spanish
ASHP: American Society of Hospital Pharmacists
NIOSH: National Institute for Occupational Safety and Health
DeCS: Descriptors in Health Sciences
BIREME: Latin American and Caribbean Center for Medical Sciences Information
MeSH: Medical Subject Headings
PRISMA: Preferred Reporting Items for Systematic Reviews and Meta-Analyses
STROBE: Strengthening the Reporting of OBservational studies in Epidemiology
SIGN: Scottish Intercollegiate Guidelines Network Grading Review Group
BK: Burton-Kebler
IP: price index
REBA: evidence-based adherence rate
