# Supplementary material for: Computer programs used in the field of hospital pharmacy for the management of dangerous drugs: systematic review of literature
Source: Front Public Health. 2023 Aug 30;11:1233264. doi: 10.3389/fpubh.2023.1233264 (PMC10498460; doi:10.3389/fpubh.2023.1233264)
Supplement: Supplementary file 1 [file Table_1.DOCX]

**Annex 1: Final equations, derived from the one used in MEDLINE, used in each of the other bibliographic databases consulted**

**Embase**

**Equation 1 - Dangerous drug**

'antineoplastic agent'/exp OR 'anti cancer drug' OR 'anti neoplastic agent' OR 'anticancer agent' OR 'anticancer drug' OR 'anticancerogen' OR 'anticarcinogen' OR 'anticarcinogenic agents' OR 'antineoplastic agent' OR 'antineoplastic agents' OR 'antineoplastic agents, combined' OR 'antineoplastic agents, phytogenic' OR 'antineoplastic combined chemotherapy protocols' OR 'antineoplastic drug' OR 'antineoplastic peptide' OR 'antineoplastics' OR 'antineoplastics, enzymes' OR 'antineoplastics, miscellaneous' OR 'antineoplastics, radiopharmaceuticals' OR 'antineoplastics, signal transduction inhibitors' OR 'antitumor agent' OR 'antitumor drug' OR 'antitumour agent' OR 'antitumour drug' OR 'cancer chemotherapeutic agent' OR 'cancer inhibitor' OR 'carcinostatic drug' OR 'drug, antineoplastic' OR 'tumor inhibitor' OR 'tumour inhibitor' OR 'dangerous goods'/exp OR 'dangerous goods' OR 'hazardous chemical' OR 'hazardous material' OR 'hazardous substances' OR 'cytostatic agent'/exp OR 'cytostatic' OR 'cytostatic agent' OR 'cytostatic agents' OR 'cytostatic drug' OR 'cytostatic drugs' OR 'cytostatic factor' OR 'cytostatics' OR 'cytostaticum'

**Equation 2 - Medical Informatics Applications**

'medical informatics'/exp OR 'american recovery and reinvestment act' OR 'clinical informatics' OR 'clinical information science' OR 'clinical information technology' OR 'health informatics' OR 'health information science' OR 'health information technology' OR 'medical computer science' OR 'medical data processing' OR 'medical informatics' OR 'medical informatics applications' OR 'medical informatics computing' OR 'medical information science' OR 'medical information technology' OR 'public health informatics' OR 'mobile application'/exp OR 'mobile app' OR 'mobile application' OR 'mobile applications' OR 'mobile apps' OR 'portable software app' OR 'portable software application' OR 'portable software applications' OR 'portable software apps' OR 'tablet application'

**Equation 3 - Hospital Pharmacy**

'hospital pharmacy'/exp OR 'clinical pharmacy service' OR 'hospital pharmaceutic service' OR 'hospital pharmaceutical service' OR 'hospital pharmaceutical services' OR 'hospital pharmacies' OR 'hospital pharmacy' OR 'hospital pharmacy service' OR 'pharmacy service, hospital'

**Cochrane Library**

**Equation 1 - Dangerous drug**

MeSH descriptor: [Antineoplastic Agents] explode all trees OR (“Antineoplastic Agents” OR “Antineoplastic Drug” OR “Antineoplastic” OR “Chemotherapeutic Anticancer Drug” OR “Antitumor Drug” OR “Cancer Chemotherapy Agent” OR “Cancer Chemotherapy Drug” OR “Chemotherapeutic Anticancer Agent” OR “Anticancer Agent” OR “Antitumor Agent”):ti,ab,kw OR MeSH descriptor: [Hazardous Substances] explode all trees OR (“Hazardous Substances” OR “Hazardous Material” OR “Hazardous Chemical” OR “Environmental Toxic Substance” OR “Toxic Environmental Substance” OR “Biohazard”):ti,ab,kw OR MeSH descriptor: [Cytostatic Agents] explode all trees OR (“Cytostatic Agents” OR “Cytostatic” OR “Cytostatic Drug” OR “Hazardous Drug” OR “Chemotherapy” OR “Chemotherapeutic Agent” OR “Chemotherapeutic Drug” OR “Antineoplastic Medication” OR “Anticancer Drug” OR “Higlhly Potent Drug”):ti,ab,kw

**Equation 2 - Medical Informatics Applications**

MeSH descriptor: [Medical Informatics Applications] explode all trees OR (“Medical Informatics Application” OR “Online System” OR “Clinical Informatic” OR “Health Informatic” OR “Medical Data Processing” OR “Medical Informatic” OR “Medical Informatics Computing” OR “Public Health Informatic”):ti,ab,kw OR MeSH descriptor: [Mobile Applications] explode all trees OR (“Mobile Application” OR “Mobile App” OR “Portable Electronic App” OR “Portable Electronic Application” OR “Portable Software App” OR “Portable Software Application” OR “Tablet Application” OR MeSH descriptor: [Software] explode all trees OR (“Computer Software” OR “Computer Program” OR “Software Tool” OR “Software Engineering” OR “Computer Applications Software” OR “Computer Software Application”):ti,ab,kw OR MeSH descriptor: [Software Design] explode all trees OR (“Software Design”):ti,ab,kw

**Equation 3 - Hospital Pharmacy**

MeSH descriptor: [Pharmacy Service, Hospital] explode all tres OR (“Hospital Pharmacy Service” OR “Hospital Pharmaceutical Service” OR “Clinical Pharmacy Service” OR “Hospital Pharmacies” OR “Hospital Pharmacy”):ti,ab,kw

**Scopus**

**Equation 1 - Dangerous drug**

TITLE-ABS-KEY (“Antineoplastic Agents” OR “Antineoplastic Drug” OR “Antineoplastic” OR “Chemotherapeutic Anticancer Drug” OR “Antitumor Drug” OR “Cancer Chemotherapy Agent” OR “Cancer Chemotherapy Drug” OR “Chemotherapeutic Anticancer Agent” OR “Anticancer Agent” OR “Antitumor Agent” OR “Hazardous Substances” OR “Hazardous Material” OR “Hazardous Chemical” OR “Environmental Toxic Substance” OR “Toxic Environmental Substance” OR “Biohazard” OR “Cytostatic Agents” OR “Cytostatic” OR “Cytostatic Drug” OR “Hazardous Drug” OR “Chemotherapy” OR “Chemotherapeutic Agent” OR “Chemotherapeutic Drug” OR “Antineoplastic Medication” OR “Anticancer Drug” OR “Higlhly Potent Drug”)

**Equation 2 - Medical Informatics Applications**

TITLE-ABS-KEY (“Medical Informatics Applications” OR “Medical Informatics Application” OR “Online System” OR “Clinical Informatic” OR “Health Informatic” OR “Medical Data Processing” OR “Medical Informatic” OR “Medical Informatics Computing” OR “Public Health Informatic” OR “Mobile Applications” OR “Mobile Application” OR “Mobile App” OR “Portable Electronic App” OR “Portable Electronic Application” OR “Portable Software App” OR “Portable Software Application” OR “Tablet Application” OR “Software” OR “Computer Software” OR “Computer Program” OR “Software Tool” OR “Software Engineering” OR “Computer Applications Software” OR “Computer Software Application” OR “Software Design”)

**Equation 3 - Hospital Pharmacy**

TITLE-ABS-KEY (“Pharmacy Service, Hospital” OR “Hospital Pharmacy Service” OR “Hospital Pharmaceutic Service” OR “Hospital Pharmaceutical Service” OR “Clinical Pharmacy Service” OR “Hospital Pharmacies” OR “Hospital Pharmacy”)

**Web of Science**

**Equation 1 - Dangerous drug**

(“Antineoplastic Agents” OR “Antineoplastic Drug” OR “Antineoplastic” OR “Chemotherapeutic Anticancer Drug” OR “Antitumor Drug” OR “Cancer Chemotherapy Agent” OR “Cancer Chemotherapy Drug” OR “Chemotherapeutic Anticancer Agent” OR “Anticancer Agent” OR “Antitumor Agent” OR “Hazardous Substances” OR “Hazardous Material” OR “Hazardous Chemical” OR “Environmental Toxic Substance” OR “Toxic Environmental Substance” OR “Biohazard” OR “Cytostatic Agents” OR “Cytostatic” OR “Cytostatic Drug” OR “Hazardous Drug” OR “Chemotherapy” OR “Chemotherapeutic Agent” OR “Chemotherapeutic Drug” OR “Antineoplastic Medication” OR “Anticancer Drug” OR “Higlhly Potent Drug”) (Topic)

**Equation 2 - Medical Informatics Applications**

(“Medical Informatics Applications” OR “Medical Informatics Application” OR “Online System” OR “Clinical Informatic” OR “Health Informatic” OR “Medical Data Processing” OR “Medical Informatic” OR “Medical Informatics Computing” OR “Public Health Informatic” OR “Mobile Applications” OR “Mobile Application” OR “Mobile App” OR “Portable Electronic App” OR “Portable Electronic Application” OR “Portable Software App” OR “Portable Software Application” OR “Tablet Application” OR “Software” OR “Computer Software” OR “Computer Program” OR “Software Tool” OR “Software Engineering” OR “Computer Applications Software” OR “Computer Software Application” OR “Software Design”) (Topic)

**Equation 3 - Hospital Pharmacy**

(“Pharmacy Service, Hospital” OR “Hospital Pharmacy Service” OR “Hospital Pharmaceutic Service” OR “Hospital Pharmaceutical Service” OR “Clinical Pharmacy Service” OR “Hospital Pharmacies” OR “Hospital Pharmacy”) (Topic)

**Latin American & Caribbean Health Sciences Literature (LILACS)**

**Equation 1 - Dangerous drug**

“Antineoplastic Agents”[Subject descriptor] OR “Antineoplastic Agent*”[Abstract Words] OR “Antineoplastic Drug*”[Abstract Words] OR “Antineoplastic*”[Abstract Words] OR “Chemotherapeutic Anticancer Drug*”[Abstract Words] OR “Antitumor Drug*”[Abstract Words] OR “Cancer Chemotherapy Agent*”[Abstract Words] OR “Cancer Chemotherapy Drug*”[Abstract Words] OR “Chemotherapeutic Anticancer Agent*”[Abstract Words] OR “Anticancer Agent*”[Abstract Words] OR “Antitumor Agent*”[Abstract Words] OR “Hazardous Substances”[Subject descriptor] OR “Hazardous Material*”[Abstract Words] OR “Hazardous Chemical*”[Abstract Words] OR “Environmental Toxic Substance*”[Abstract Words] OR “Toxic Environmental Substance*”[Abstract Words] OR “Biohazard*”[Abstract Words] OR “Cytostatic Agents”[Subject descriptor] OR “Cytostatic Agents*”[Abstract Words] OR “Cytostatic*”[Abstract Words] OR “Cytostatic Drug*”[Abstract Words] OR “Hazardous Drug*”[Abstract Words] OR “Chemotherapy”[Abstract Words] OR “Chemotherapeutic Agent*”[Abstract Words] OR “Chemotherapeutic Drug*”[Abstract Words] OR “Antineoplastic Medication*”[Abstract Words] OR “Anticancer Drug*”[Abstract Words] OR “Higlhly Potent Drug*”[Abstract Words]

**Equation 2 - Medical Informatics Applications**

“Medical Informatics Applications”[Subject descriptor] OR “Medical Informatics Application*”[Abstract Words] OR “Online System*”[Abstract Words] OR “Clinical Informatic*”[Abstract Words] OR “Health Informatic*”[Abstract Words] OR “Medical Data Processing”[Abstract Words] OR “Medical Informatic*”[Abstract Words] OR “Medical Informatics Computing”[Abstract Words] OR “Public Health Informatic*”[Abstract Words] OR “Mobile Applications”[Subject descriptor] OR “Mobile Application*”[Abstract Words] OR “Mobile App*”[Abstract Words] OR “Portable Electronic App*”[Abstract Words] OR “Portable Electronic Application*”[Abstract Words] OR “Portable Software App*”[Abstract Words] OR “Portable Software Application*”[Abstract Words] OR “Tablet Application*”[Abstract Words] OR “Software”[Mesh] OR “Computer Software”[Abstract Words] OR “Computer Program*”[Abstract Words] OR “Software Tool*”[Abstract Words] OR “Software Engineering*”[Abstract Words] OR “Computer Applications Software*”[Abstract Words] OR “Computer Software Application*”[Abstract Words] OR “Software Design”[Subject descriptor] OR “Software Design”[Abstract Words]

**Equation 3 - Hospital Pharmacy**

“Pharmacy Service, Hospital”[Subject descriptor] OR “Hospital Pharmacy Service*”[Abstract Words] OR “Hospital Pharmaceutic Service*”[Abstract Words] OR “Hospital Pharmaceutical Service*”[Abstract Words] OR “Clinical Pharmacy Service*”[Abstract Words] OR “Hospital Pharmacies”[Abstract Words] OR “Hospital Pharmacy”[Abstract Words]

**Medicina en Español (MEDES)**

**Equation 1 - Dangerous drug**

(“Agentes antineoplásicos”[título/resumen/palabras_clave]) O (“Medicamento antineoplásico”[título/resumen/palabras_clave]) O (“Antineoplásico”[título/resumen/palabras_clave]) O (“Medicamento quimioterapéutico contra el cáncer”[título/resumen/palabras_clave]) O (“Medicamento antitumoral”[título/resumen/palabras_clave]) O (“Agente de quimioterapia contra el cáncer”[título/resumen/palabras_clave]) O (“Medicamento de quimioterapia contra el cáncer”[título/resumen/palabras_clave]) O (“Agente quimioterapéutico contra el cáncer”[título/resumen/palabras_clave]) O (“Agente contra el cáncer”[título/resumen/palabras_clave]) O (“Agente antitumoral”[título/resumen/palabras_clave]) O (“Sustancias peligrosas”[título/resumen/palabras_clave]) O (“Material peligroso”[título/resumen/palabras_clave]) O (“Químico peligroso”[título/resumen/palabras_clave]) O (“Sustancia tóxica ambiental”[título/resumen/palabras_clave]) O (“Sustancia ambiental tóxica”[título/resumen/palabras_clave]) O (“Riesgo biológico”[título/resumen/palabras_clave]) O (“Agentes citostáticos”[título/resumen/palabras_clave]) O (“Citostático”[título/resumen/palabras_clave]) O (“Fármaco citostático”[título/resumen/palabras_clave]) O (“Medicamento peligroso”[título/resumen/palabras_clave]) O (“Quimioterapia”[título/resumen/palabras_clave]) O (“Agente quimioterapéutico”[título/resumen/palabras_clave]) O (“Medicamento quimioterapéutico”[título/resumen/palabras_clave]) O (“Medicamento antineoplásico”[título/resumen/palabras_clave]) O (“Medicamento contra el cáncer”[título/resumen/palabras_clave]) O (“Medicamento altamente potente”[título/resumen/palabras_clave])

**Equation 2 - Medical Informatics Applications**

(“Aplicaciones de informática médica”[título/resumen/palabras_clave]) O (“Aplicación de informática médica”[título/resumen/palabras_clave]) O (“Sistema en línea”[título/resumen/palabras_clave]) O (“Informática clínica”[título/resumen/palabras_clave]) O (“Informática de salud”[título/resumen/palabras_clave]) O (“Procesamiento de datos médicos”[título/resumen/palabras_clave]) O (“Informática médica”[título/resumen/palabras_clave]) O (“Informática de salud pública”[título/resumen/palabras_clave]) O (“Aplicaciones móviles”[título/resumen/palabras_clave]) O (“Aplicación móvil”[título/resumen/palabras_clave]) O (“Aplicación electrónica portátil”[título/resumen/palabras_clave]) O (“Aplicación de software portátil”[título/resumen/palabras_clave]) O (“Tableta Aplicación”[título/resumen/palabras_clave]) O (“Software”[título/resumen/palabras_clave]) O (“Software Informático”[título/resumen/palabras_clave]) O (“Programa Informático”[título/resumen/palabras_clave]) O (“Herramienta De Software”[título/resumen/palabras_clave]) O (“Ingeniería De Software”[título/resumen/palabras_clave]) O (“Software De Aplicaciones Informáticas”[título/resumen/palabras_clave]) O (“Aplicación De Software Informático”[título/resumen/palabras_clave]) O (“Diseño De Software”[título/resumen/palabras_clave]**)**

**Equation 3 - Hospital Pharmacy**

(“Servicio de Farmacia Hospitalaria”[título/resumen/palabras_clave]) O (“Servicios de Farmacia Hospitalaria”[título/resumen/palabras_clave]) O (“Servicio de Farmacia Clínica”[título/resumen/palabras_clave]) O (“Farmacias Hospitalarias”[título/resumen/palabras_clave]) O (“Farmacia Hospitalaria”[título/resumen/palabras_clave])
